# Predicting bone strength with ultrasonic guided waves

**DOI:** 10.1038/srep43628

**Published:** 2017-03-03

**Authors:** Nicolas Bochud, Quentin Vallet, Jean-Gabriel Minonzio, Pascal Laugier

**Affiliations:** 1 Sorbonne Universités, UPMC Univ Paris 06, CNRS UMR 7371, INSERM UMR S1146, Laboratoire d’imagerie biomédicale, 15 rue de l’école de médecine, F-75006, Paris, France

## Abstract

Recent bone quantitative ultrasound approaches exploit the multimode waveguide response of long bones for assessing properties such as cortical thickness and stiffness. Clinical applications remain, however, challenging, as the impact of soft tissue on guided waves characteristics is not fully understood yet. In particular, it must be clarified whether soft tissue must be incorporated in waveguide models needed to infer reliable cortical bone properties. We hypothesize that an inverse procedure using a free plate model can be applied to retrieve the thickness and stiffness of cortical bone from experimental data. This approach is first validated on a series of laboratory-controlled measurements performed on assemblies of bone- and soft tissue mimicking phantoms and then on *in vivo* measurements. The accuracy of the estimates is evaluated by comparison with reference values. To further support our hypothesis, these estimates are subsequently inserted into a bilayer model to test its accuracy. Our results show that the free plate model allows retrieving reliable waveguide properties, despite the presence of soft tissue. They also suggest that the more sophisticated bilayer model, although it is more precise to predict experimental data in the forward problem, could turn out to be hardly manageable for solving the inverse problem.

Osteoporosis is a medical threat with a consequent increase in bone fragility and susceptibility to fracture. There is an increasing awareness about osteoporosis, because of the consequences of fractures on morbidity, quality of life and mortality^1^. The standard approach for the diagnosis of osteoporosis and fracture risk prediction is based on the use of ionizing dual energy X-ray absorptiometry (DXA) in order to assess bone mineral density (BMD). The role of cortical bone as a determinant of bone strength was recently highlighted^2^,^3^,^4^, as a result of which it has been suggested that diagnosis (risk assessment) should include accurate evaluation of cortical bone^5^. However, the structural properties (i.e., thickness and porosity) of cortical bone are poorly captured in patients by X-ray densitometry or computed tomography techniques. Furthermore, the non-invasive *in vivo* assessment of its material properties (i.e., stiffness) remains unattainable to date. In response to these issues, sophisticated quantitative ultrasound (QUS) approaches are currently being explored, based on the experimental evidence that cortical bone supports propagation of multiple guided waves (GWs)^6^,^7^. GWs are dispersive and the relation between the wave number (*k*) and frequency (*f*), specific to each guided mode, is determined by the geometric and elastic properties of the waveguide. Changes in cortical thickness and elastic properties observed with aging, osteoporosis or in response to treatments are expected to change the propagation characteristics of GWs. Consequently, GWs measurements on long bones such as the radius, along with appropriate waveguide modeling, have the potential for yielding strenght-related bone properties that can be used as biomarkers to identify patients at risk of fracture or to judge whether a patient has responded well to treatment.

At the macro-scale, the cortical shell of human long bones is an absorbing and anisotropic material, whose geometry is that of an irregular hollow structure filled with marrow and surrounded by soft tissue. The suitability of the waveguide model used to retrieve *in vivo* cortical bone properties from GWs measurements is a key step in data processing. Here, the purpose of the waveguide model is to represent a simplified but sufficient model that fits the experimental ultrasound data and provides a robust inference of one or more waveguide properties. The question arises as to whether the waveguide model must include the effects of soft tissue coating, bone curvature and irregular geometry, anisotropy and some other additional factors as well, such as, e.g., the absorption. Given the complexity of the bone waveguide, the choice of a model arises from the trade-off between the complexity of the model (i.e., number of model parameters) and the accuracy of the estimates (i.e., stability of the inverse problem solution in terms of convergence, existence and uniqueness). Several phantom and *ex vivo* studies focused on such GWs model-based approaches. Among these, authors reported estimates of cortical thickness (assuming a fixed elasticity) using an isotropic hollow cylinder model^8^ or an isotropic hollow cylinder model filled with viscous liquid^9^,^10^. In another related study, authors reported estimates of Young’s modulus (assuming a fixed thickness), using an isotropic plate model^7^. More recently, our group achieved concurrent estimates of both thickness and elastic properties of cortical bone using a transverse isotropic plate model^11^. In all these studies, only non-absorbing waveguides were considered. Hence, the outcomes of these earlier studies suggested that relatively simple models can deliver convincing results.

Clinical applications using GWs remain, however, challenging for assessing multiple bone biomarkers (e.g., cortical thickness, stiffness and porosity), as the impact of the soft tissue layer on top of bone is not well established yet. The effect of the soft tissue layer may be twofold. First, the soft tissue layer nearly behaves like a fluid waveguide for ultrasound, which can give rise to additional guided modes^12^,^13^. These modes propagating in soft tissue may be mistaken with those propagating in bone when their velocities overlap^14^,^15^. A second effect arises from the coupling of the two waveguides (i.e., the soft tissue and the bone waveguides), potentially resulting in a modification of the dispersion curves of the bone waveguide^16^. To date, the influence of overlying soft tissue has only been analyzed in a few phantom studies^12^,^13^,^14^,^15^,^17^,^18^,^19^,^20^ (see Supplementary Introduction and Table S1). The main conclusions that can be drawn from these studies are as follows: (1) the presence of soft tissue attenuates the time-domain signals, (2) the number of modes significantly increases with increasing soft tissue thickness, and (3) the dispersion curves belonging to the solid waveguide are only modified in a certain range of the *f*−*k* plane. A recent study by our group reported the first *in vivo* estimates of cortical thickness in healthy subjects using multimode GWs measurements^21^. Nonetheless, this study assumed a fixed elasticity and only considered higher-order modes (i.e., phase velocity higher than 3 mm·*μ*s^−1^) to avoid the influence of soft tissue, in which case a simple plate model provided an appropriate inverse model. It has also been shown that the use of sophisticated models (e.g., uncoupled bilayer^12^, fluid-solid bilayer^15^, solid-solid bilayer^13^, three-layered system^19^) can explain additional data arising from the presence of soft tissue mimics. These studies, however, only solved the forward problem using *a priori* known bone-mimicking properties, without providing any hints on a possible inversion, i.e., on the ability to infer properties of cortical bone for clinical purposes. It is not clear yet that such sophisticated models could be used *in vivo*, where both cortical bone and soft tissue properties are unknown, and experimental data are noisy and incomplete. In addition, none of these studies except^12^,^19^ considered bone as transverse isotropic, whereas it is expected that anisotropy has a significant influence for recovering reliable estimates of cortical bone properties^22^,^23^.

Following these studies, no general consensus has been reached regarding the complexity of the waveguide model to be used to recover reliable estimates of cortical bone properties *in vivo*. Consequently, the present study aims at determining to which extent a two-dimensional (2-D) transverse isotropic free plate waveguide model is accurate enough for this purpose, despite the presence of soft tissue and bone curvature. The choice of this model is based on earlier findings that dealt with a similar frequency-thickness product range^12^,^20^, which evidenced that the additional modes due to the presence of overlying soft tissue mimics do not significantly modify the trajectory of the modes belonging to the solid (bone-mimicking phantom) subsystem, and can thus be predicted by a free plate model. We hypothesized here that the same conclusion holds for *in vivo* measurements, i.e., that the dispersion curves obtained *in vivo* contain data that exhibit the fingerprints of the cortical bone subsystem that are not significantly affected by the soft tissue, and that can be reasonably approximated by a free plate model. To test this hypothesis within the context of multimode GWs measurements (i.e., large frequency (*f*) - thickness (*h*_*s*_) product), we propose a two-step approach. In a first step, we perform an inversion of both the thickness and elastic properties of different waveguide structures using a 2-D transverse isotropic free plate model. The inverse problem is solved using an efficient model-based approach recently developed by our group^24^, and the accuracy of the inferred estimates is evaluated by comparison with reference values. In a second step, as reference values for *in vivo* cortical bone assessment are typically unavailable, the inferred estimates are subsequently inserted into a 2-D free bilayer model to test if outliers (i.e., experimental data that are not explained with the free plate model) are fitted properly. This two-steps approach is first validated on a series of laboratory-controlled measurements performed on assemblies of soft tissue- and bone-mimicking phantoms (plates and tubes), whose thickness and material properties cover a representative range of expected cortical bone properties. Then, this approach is illustrated on a few *in vivo* measurements at the forearm.

## Methods

### Experimental measurements

#### Samples and reference measurements

Laboratory-controlled GWs measurements were carried out on twenty different bilayer assemblies, which result from the combination of bone-mimicking plates or tubes coated with layers of soft tissue-mimicking phantoms. The bone-mimicking material is a transverse isotropic composite made of short glass fibers embedded in an epoxy matrix (Sawbones^®^, Pacific Research Laboratories Inc., Vashon Island). The plates and tubes thickness ranged from 1 to 4 millimeters, which is typical for the cortical thickness of human bones^11^,^15^. The tubes had a transverse circular cross-section with an external radius of curvature of 17.5 millimeters. The reference thickness of the plates and tubes was directly derived from caliper measurements, repeated five times in the area of GWs measurements. Reference stiffness coefficients were obtained by performing resonant ultrasound spectroscopy (RUS) measurements, as RUS currently represents the reference technique for measuring the anisotropic elasticity of solid materials^25^. Samples for RUS measurements were prepared by cutting small-sized “parallelepiped-shaped” specimens from the volume of the coated plates and tubes measured by GWs. The mass density of each sample was deduced from a measurement of the dimensions using a digital caliper (accuracy of 0.01 mm) and a mass measurement (accuracy of 0.1 mg). For each sample, the resonant frequencies of the freely vibrating solid of known shape and mass density were measured and an iterative procedure was used to adjust the stiffness coefficients until the numerically predicted spectrum matched the measured frequencies^26^.

The mean and standard deviation of the resulting reference stiffness coefficients and mass density are listed in the first row of Table 1.

The materials of the soft tissue-mimicking phantoms are solid water-based polymers (Zerdine and Urethane, CIRS, Norfolk, Virginia, USA). The structural and acoustic properties of these two materials, provided by the manufacturer, are listed in Table 2. These soft tissue-mimicking phantoms were chosen owing to the similarity of their acoustic properties with those of human soft tissue. Their thickness ranged from 2 to 10 millimeters, which is consistent with the soft tissue thickness that can be expected at the third from the distal end of the forearm^15^.

In addition, four healthy volunteers recruited from the laboratory staff (three males and one female, aged from 27 to 51 years old) were measured at the third from the distal end of the radius by positioning the center of the probe approximately 70 mm away from the radial styloid^21^. The local ethics committee (Comité de protection des personnes Ile de France III) approved the protocol and informed consent was obtained from all subjects in accordance with regulations. The length of the region of interest, prescribed by the length of the receiver array, was 20 mm on the postero-lateral face of the forearm. Reference measurements were performed independently of the GWs measurements to assess the correctness of the ultrasound-based estimates. Reference cortical thickness values were delivered by site-matched high-resolution X-ray peripheral computed tomography (HR-pQCT) measurements (XtremCT, Scanco Medical, Brutisellen, Switzerland) with a voxel size of 82 *μ*m (spatial resolution of 128 *μ*m). All methods were performed in accordance with the relevant guidelines and regulations. The thickness of the soft tissue was estimated by performing standard 20 MHz pulse-echo measurements (see Table 2), ensuring no probe loading to avoid deforming soft tissue. Stiffness coefficients and mass density were compared to typical range of values from the literature^27^,^28^,^29^ (see second row of Table 1).

#### Experimental set-up and extraction of the dispersion curves

GWs measurements were performed in axial transmission configuration with a custom-made probe (Vermon, Tours, France), which consists of a 24 elements receiving array surrounded by two arrays of 5 emitters each. The three arrays of piezocomposite elements, aligned along the *x*_3_-axis (i.e., main fibers orientation for the bone-mimicking phantoms and main bone axis *in vivo*), were in contact with the samples and ultrasonic gel was used for coupling. This configuration permits the propagation of GWs in two opposite directions, thus allowing the correction of the bias induced by the eventual inclination between the probe and the samples, which could result from the presence of uneven overlying soft tissue in the measurements area^30^.

A custom-made electronic device (Althaïs Technologies, Tours, France) was used to transmit wideband ultrasonic pulses at a central frequency of 1 MHz (−6 dB power spectrum spanning the frequency range from 0.4 to 1.6 MHz) and to record the received signals. For each propagation direction, a set of 5 × 24 = 120 radio-frequency signals corresponding to all possible pairs of emitter/receiver elements were digitized (12 bits, 20 MHz, 1024 samples) after 16 averages by hardware.

The procedure to extract the experimental dispersion curves, representing the frequency-dependent wave numbers (i.e., *k(f*)) of guided modes propagating in the waveguide, was straightforwardly applied following^21^,^31^.

In short, (1) for each propagation direction, the 120 radio-frequency signals were Fourier transformed with respect to time and stored in a response matrix; (2) a singular value decomposition was applied to the response matrix at each frequency; (3) signal-to-noise ratio enhancement was achieved by removing the singular vectors corresponding to the lowest singular values; (4) a testing vector (i.e., an attenuated spatial plane wave with a complex wave number^32^) was projected onto the singular vector basis, delivering the so-called *Norm function*, whose maxima correspond to the wave numbers of the guided modes; (5) combining the data acquired from the two propagation directions, a bidirectional correction was applied to the measurements^30^; (6) the (*f, k*)–pairs were extracted from the corrected *Norm function* using a dilation operator^21^; (7) outliers were removed from those pairs by applying statistical denoising over 10 measurement repetitions (successively recorded without moving the probe); and (8) the resulting denoised data on each direction were grouped together, yielding a single set of dispersion curves for each sample. For further details on the data processing, the reader is referred to our previous studies^21^,^31^,^32^.

**Figure 1:**
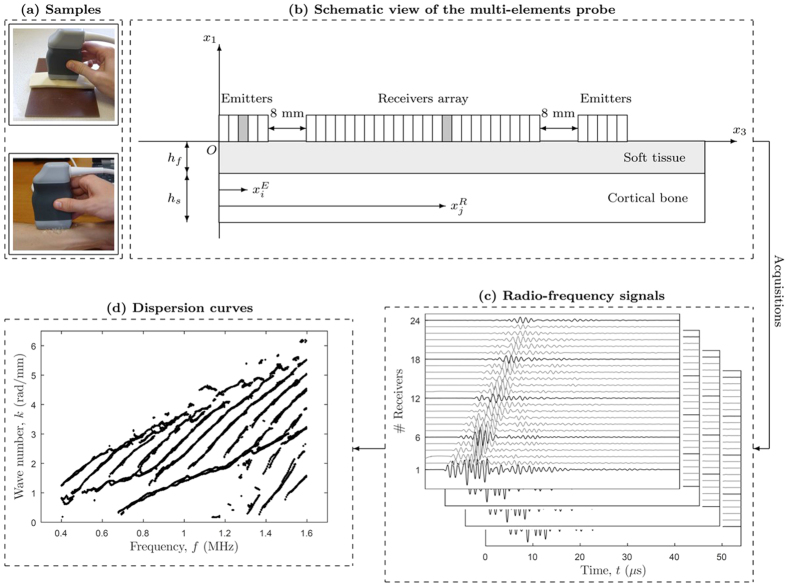
Overview of the experimental setup: (**a**) Pictures of the custom-made probe in contact with a bilayer phantom and the forearm of a subject; (**b**) Schematic view of the multi-elements probe, which consists of a 24 elements receiving array surrounded by two arrays of 5 emitters each, aligned along the main bone axis *x*_3_; (**c**) acquired radio-frequency signals for each pair of emitter-receiver; and (**d**) extracted experimental dispersion curves after applying the signal processing steps.

Figure 1 depicts an example of some of the investigated specimens, along with the experimental setup used to obtain the dispersion curves.

### Forward calculation of the dispersion curves

Two waveguide models were used in the present study to fit the experimental dispersion curves. The first model, a free plate model, was used to test the reliability of such simplistic model for inferring estimates of cortical bone properties by solving a model-based inverse problem^24^. The second model, a fluid-solid bilayer model, was used to further support these estimates by solving the forward problem.

#### 2-D free plate model

In this paper, the choice of the waveguide model is based on the following assumptions derived from earlier observations: (i) at 1 MHz, ultrasound waves are sensitive to the effective (i.e., mesoscopic) elastic properties of bone, which can thus be considered as a homogeneous material^29^,^33^; (ii) the cortical thickness can be assumed as constant in the region prescribed by the length of the receiver array^34^; and (iii) given our probe configuration and the driving-frequency, the tubular bone shape can be locally approximated by a plate despite bone curvature^20^. Therefore, in what follows, cortical bone is considered as a two-dimensional (2-D) transverse isotropic homogeneous free plate waveguide. For such a model, the solutions of the corresponding Lamb wave equation for propagation in the meridian plane can be expressed as guided modes in the *f*−*k* plane, which are determined by prescribed thickness *h*_*s*_ of the waveguide, mass density *ρ*_*s*_, and stiffness coefficients *c*_11_, *c*_33_, *c*_13_, and *c*_55_^11^,^12^,^35^. Alternatively, the dispersion equation can be formulated as function of the bulk wave velocities, the mass density being embedded in the velocity parameters. The resulting model parameters that account for the stiffness of the waveguide are compound of two bulk wave velocities and two stiffness ratios (containing the off-diagonal coefficient and the anisotropic ratio), defined as:


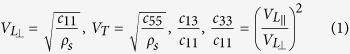


where 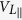, 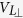, and *V*_*T*_ denote the longitudinal bulk wave velocities (along and normal to the *x*_3_-axis) and the transverse bulk wave velocity, respectively.

#### Bilayer model

The fluid-solid bilayer model has been introduced by Yapura and Kinra^16^. Initially, the dispersion equation was developed considering that the solid subsystem of the bilayer was isotropic. Subsequently, this model was extended to account for an orthotropic solid subsystem^36^. In this study, we considered an intermediary case, in which the solid subsystem was considered as transverse isotropic and non-absorbing.

Note that the fluid-transverse isotropic solid model interface may differ from that of the soft tissue and cortical bone, in that fluid does not sustain shear stresses whereas soft tissue does. Nonetheless, such difference is not believed to induce a too large deviation while modeling the guided modes of the considered waveguide structures^14^, as shear waves do not propagate in the considered ultrasonic frequency bandwidth. A fluid-solid bilayer system introduces extra constraints to the particle motion at the interface (compared with free boundary conditions), so that the energy of the GWs leaks from solid to fluid in the form of leaky waves^37^. When these waves encounter boundaries of different media, they are reflected back to the top boundary of the overlying fluid and propagate back to the solid substrate. Consequently, the GWs do not propagate in the fluid or solid layer alone (as suggested by Chen *et al*.^12^), but in the whole bilayer structure. This coupling is known to affect the dispersion characteristics of the guided modes.

The characteristic equation for such fluid-transverse isotropic solid bilayer waveguide can be written in the form,


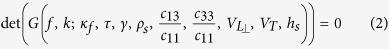


where *κ*_*f*_ = *c*_*f*_/*V*_*T*_, *τ* = *h*_*f*_/*h*_*s*_, and *γ* = *ρ*_*f*_/*ρ*_*s*_. Subscripts *s* and *f* characterize the models parameters, whether they belong to the solid layer or the fluid coating, respectively. Note that, in contrast to the plate model, the density of the solid must now be explicitly incorporated within the dispersion equation.

**Figure 2:**
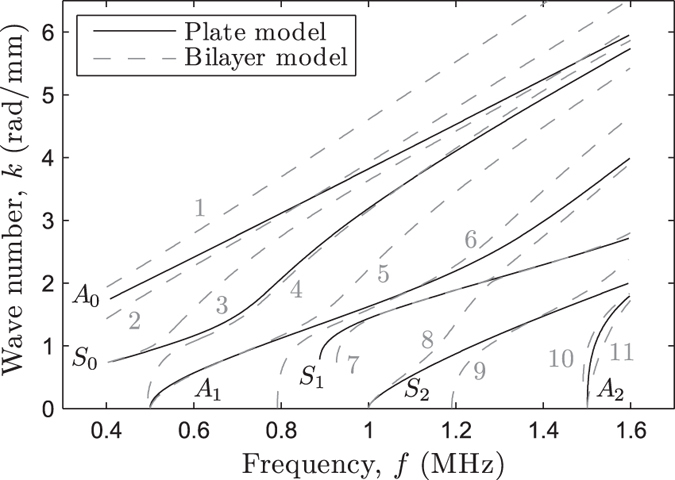
Example of the plate (continuous lines) and bilayer (dotted lines) modes for *h*_*s*_ = 1.8 mm, *κ*_*f*_ = 0.83, *τ* = 1.25, and *γ* = 0.54 (the mass density *ρ*_*s*_ and elastic coefficients *c*_*ij*_ were taken from Bossy *et al*.^22^).

An example of the plate and bilayer modes is depicted in Fig. 2. Because the notation of antisymmetric (*A*_*n*_) and symmetric (*S*_*n*_) modes is reserved for uncoated solids and the guided modes issued from a bilayer model are no longer classical Lamb modes, we follow the ordinal numbers notation by Yapura and Kinra^16^ to label the bilayer modes (*n*). As can be observed, the bilayer model generates additional modes and modifies the trajectory of the modes issued from a plate model, particularly in the area of the *f*−*k* plane where the modes reach their cut-off frequencies (*k* = 0 rad·mm^−1^) or asymptotic behavior (*f* = 1.6 MHz).

### Inverse problem

The inverse procedure used to retrieve estimates of the structural and elastic properties of waveguide structures from multimode GWs measurements has been comprehensively described in our earlier study^24^. Typically, such model-based approach comprises four elements, namely (i) the experimental data, (ii) the model parametrization, (iii) the definition of an objective function, as a metric to compare measured and modeled guided modes, and (iv) the formulation of an algorithm to iteratively optimize the objective function.

The experimental data are represented by the (*f, k*)-pairs, whose extraction has been described in a former section. The model parameters ***θ*** is a vector that consists of the waveguide thickness *h*_*s*_ (i.e., plate, tube or cortical bone), the stiffness ratios and bulk wave velocities from Eq. (1), and a pairing vector ***M***. The latter represents the combination of Lamb modes that are necessary to explain the experimental data, without requiring prior knowledge on the mode-order *n*. The objective function *F(**θ***) is defined as the *occupancy rate* of the Lamb modes^24^,


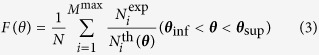


restricted to:


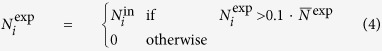


where *M*^max^ is the maximal number of modeled Lamb modes, 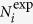 and 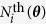 denote the number of experimental and theoretical data of a mode *i*, respectively; 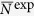 is the mean of the 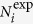; 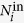 is the number of inliers of a mode *i*; and *N* is the total number of experimental data. Briefly, Eqs (3, 4) mean that experimental data can only form an experimental trajectory if a sufficiently large amount of them belong to a Lamb mode. Furthermore, an experimental data is considered as an inlier of a mode *i* if its euclidean distance *d* to that mode satisfies the following condition:


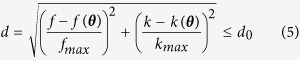


where *d*_0_ = 0.025 is a user-defined dimensionless threshold, which approximately corresponds to the normalized resolution in *k* (that is, (*π/L*)/*k*_*max*_ with *L* being the length of the receivers array)^21^,^24^. Finally, the optimal model parameters 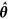 are found by maximizing the objective function *F(**θ***),


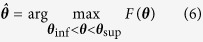


where ***θ***_inf_ and ***θ***_sup_ denote the lower and upper bounds of the model parameters ***θ***. Genetic algorithms are applied to maximize Eq. (6), due to their capability of finding a near global solution in situations where the objective function is multidimensional and multimodal^38^. The bounds [***θ***_inf_ ***θ***_sup_] of the model parameters ***θ*** that define the problem domain were set according to^11^,^24^, so that they encompass realistic values for both the bone-mimicking phantoms and cortical bone: *h*_*s*_ ∈ [0.5 4], *c*_13_/*c*_11_ ∈ [0.2 0.7], *c*_33_/*c*_11_ ∈ [1.1 2.5], 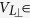
[1.63 7], and *V*_*T*_ ∈ [1.0 2.0].

## Results

### Bilayer phantoms

**Figure 3:**
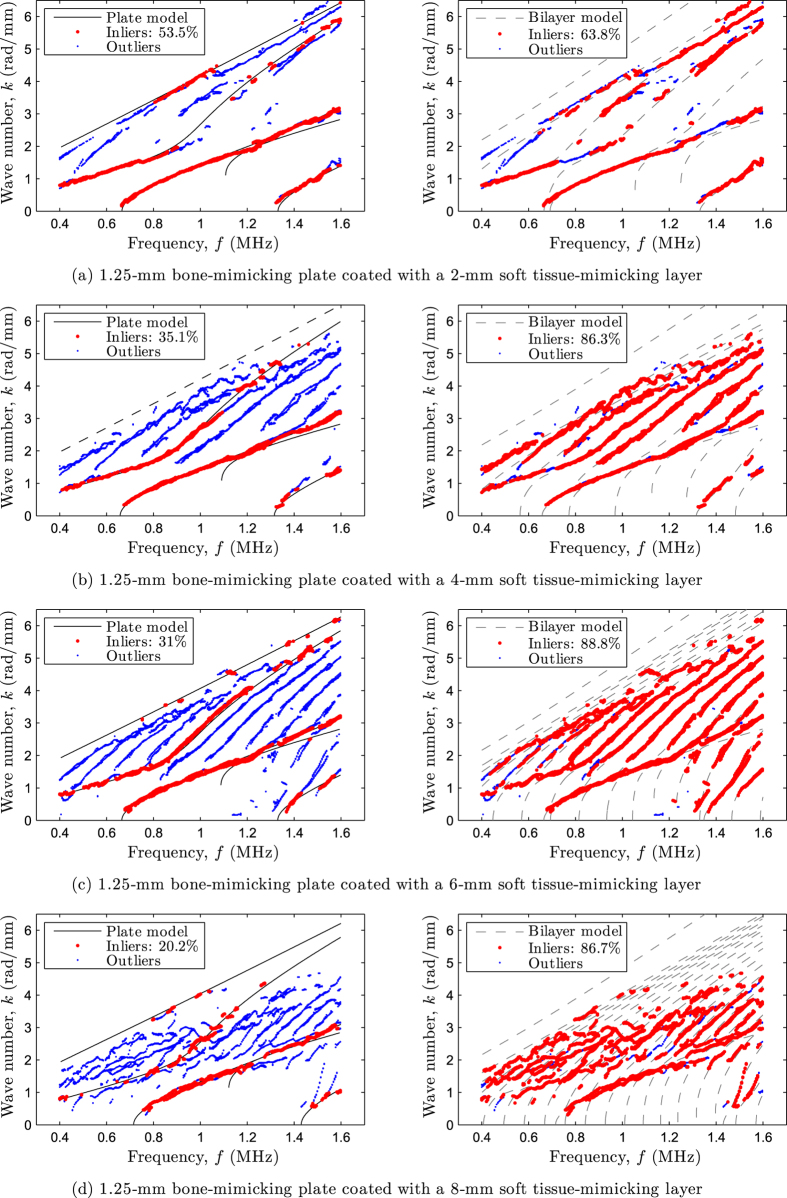
Matching between experimental data (dots) and theoretical modes (lines) for a 1.25 mm thick bone-mimicking plate coated with soft tissue-mimicking layers of increasing thickness: (**a**) 2 mm, (**b**) 4 mm, (**c**) 6 mm, and (**d**) 8 mm. Left plots represent the optimal matching resulting from the inverse problem solutions using the free plate model (modes that are missing in the optimal pairing vector ***M*** are displayed in discontinuous lines), whereas right plots represent the forward problem solutions calculated with the bilayer model.

First, results on bilayer phantoms are presented. To serve as a first example, the matching between experimental data and theoretical modes is depicted in Fig. 3 for a 1.25 mm thick bone-mimicking plate coated with soft tissue-mimicking layers of increasing thickness (2–8 mm). Each of the left plots represents the optimal matching resulting from the inverse problem solution using the free plate model. Right plots represent the forward problem solutions calculated with the bilayer model, using as input values the optimal model parameters obtained with the free plate model plus the soft tissue-mimicking properties reported in Table 2.

As can be observed in the left plots, the optimal inverse problem solutions reveal that parts of the experimental data (represented as red dots) correspond to modes belonging to the bone-mimicking subsystem (matched by the free plate modes represented as continuous lines), even in the presence of rather thick soft tissue-mimicking layers (e.g., cases (*c*)–(*d*)), which induced numerous additional modes and coupling effects explaining the increasing number of outliers (represented as blue dots). On the other hand, the forward calculation using the bilayer model (right plots) is more precise than the free plate model as it predicts most of the experimental data (see the relative percentages of inliers). Note that outliers are compound of both additional modes at low phase velocities (i.e., nearly pure fluid modes) and modified modes due to coupling effects at the interface between the soft tissue mimics and the plate (e.g., particularly observable between *f* = 1.4–1.6 MHz and *k* = 1 rad·mm^−1^ for cases (*b*)–(*d*)). Despite the complexity of the data, our model-based inverse procedure using the free plate model delivered accurate estimates of both the thickness and bulk wave velocities of the plate, as will be commented below (see Table 3).

**Figure 4:**
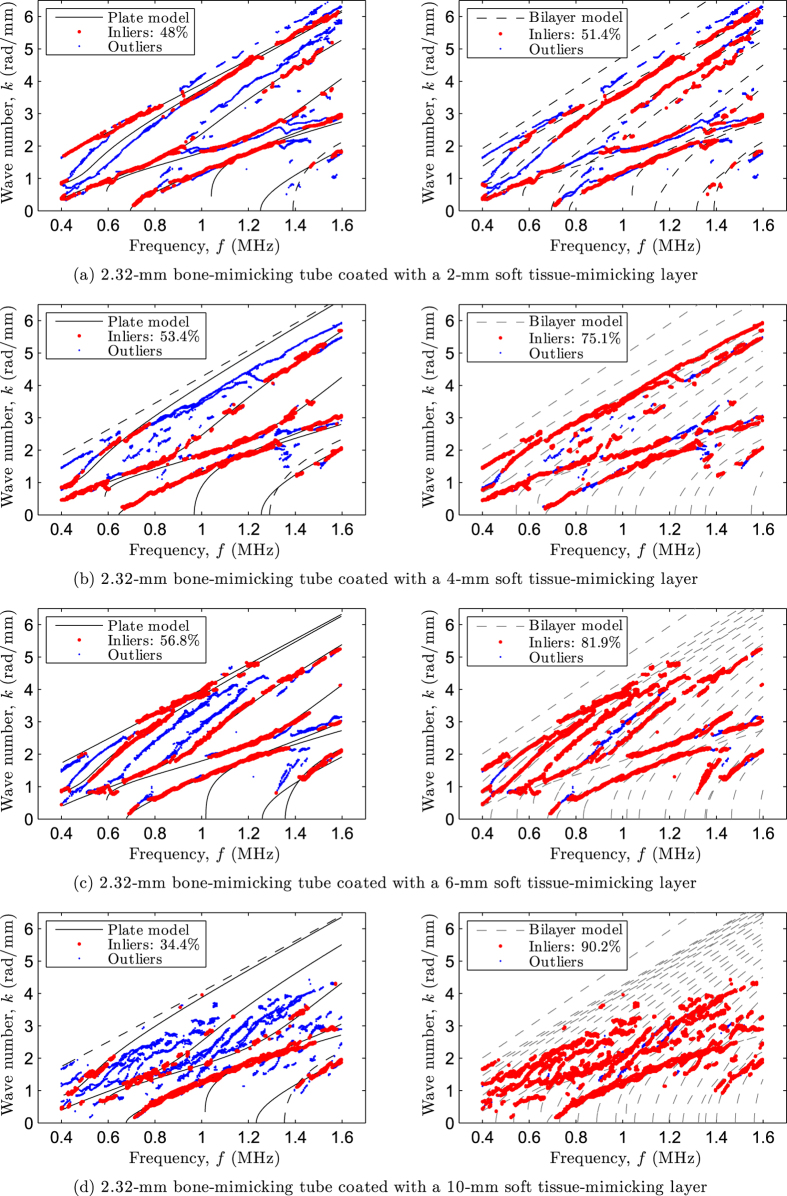
Matching between experimental data (dots) and theoretical modes (lines) for a 2.32 mm thick bone-mimicking tube coated with soft tissue-mimicking layers of increasing thickness: (**a**) 2 mm, (**b**) 4 mm, (**c**) 6 mm, and (**d**) 10 mm. Left plots represent the optimal matching resulting from the inverse problem solutions using the free plate model (modes that are missing in the optimal pairing vector ***M*** are displayed in discontinuous lines), whereas right plots represent the forward problem solutions calculated with the bilayer model.

As a second example, the matching between experimental data and theoretical modes is depicted in Fig. 4 for a 2.32 mm thick bone-mimicking tube coated with soft tissue-mimicking layers of increasing thickness (2–10 mm). As in the previous case, left plots show the inverse problem solutions using the free plate model, whereas right plots show the forward problem solutions using the bilayer model. Again, the optimal inverse problem solutions reveal that parts of the experimental data correspond to modes belonging to the bone-mimicking subsystem (matched by the free plate modes), despite the presence of soft tissue-mimicking layers and the sample curvature. The latter observation strengthens the hypothesis that, in our case, axial measurements on a coated non-flat contact surface do not significantly differ from those obtained from coated plates^20^. In addition, these results suggest that a coated tube is less affected by the presence of soft tissue than a coated plate. For instance, the relative percentages of inliers resulting from the inverse procedure are 31% and 57% for the coated plate and tube, respectively (see Figs 3(c)–4(c)).

For the additional bilayer phantoms investigated in this study (see Supplementary Figs S1–S3), the optimal inverse problem solutions also revealed that parts of the experimental data correspond to modes belonging to the bone-mimicking subsystem (matched by the free plate modes), despite the presence of soft tissue-mimicking layers and the sample curvature.Similarly, the forward calculation using the bilayer model turned out to be more accurate than the free plate model as it predicted most of the experimental data (see the relative percentages of inliers). For all the tested cases, Table 3 summarizes the ultrasound-based estimates 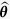, which are compared to reference values. It must be noted that the ultrasound-based approach delivers elasticity estimates by means of stiffness ratios and bulk wave velocities, while the reference method directly provides stiffness coefficients (see Table 1). Consequently, a face-to-face comparison can only be performed by making use of Eq. (1), which allows the computation of the bulk wave velocities from the reference stiffness values and mass density.

As can be observed, there is a good overall agreement between the ultrasound-based estimates and reference values. There are no significant differences between the estimates obtained for the coated plates and tubes. The relative differences on thickness range from 0% to 7% and are in the order of magnitude of the precision of the reference method. In the same way, the relative difference on bulk wave velocities are in the order of magnitude of the precision of the reference method, except for a few cases (represented in bold). These larger errors are mainly associated with bilayer phantoms with large soft tissue-mimicking thickness (i.e., 8–10 mm), for which the experimental data exhibit a rather poor *A*_0_-mode that could not be identified with the free plate model (e.g., Fig. 4(d)). Such mode is known to be mostly sensitive to the transverse bulk wave velocity component, and may thus explain the larger discrepancy on the inferred estimates of *V*_*T*_. On the other hand, for each bone-mimicking plate or tube, the variability of the estimates resulting from the presence of the soft tissue layers, calculated as the half-range divided by the median value over the four coatings, was less than 6% for *h*_*s*_, 6% for 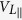, 4% for 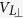, and 7% for *V*_*T*_.

### *In vivo* cortical bone assessment

**Figure 5:**
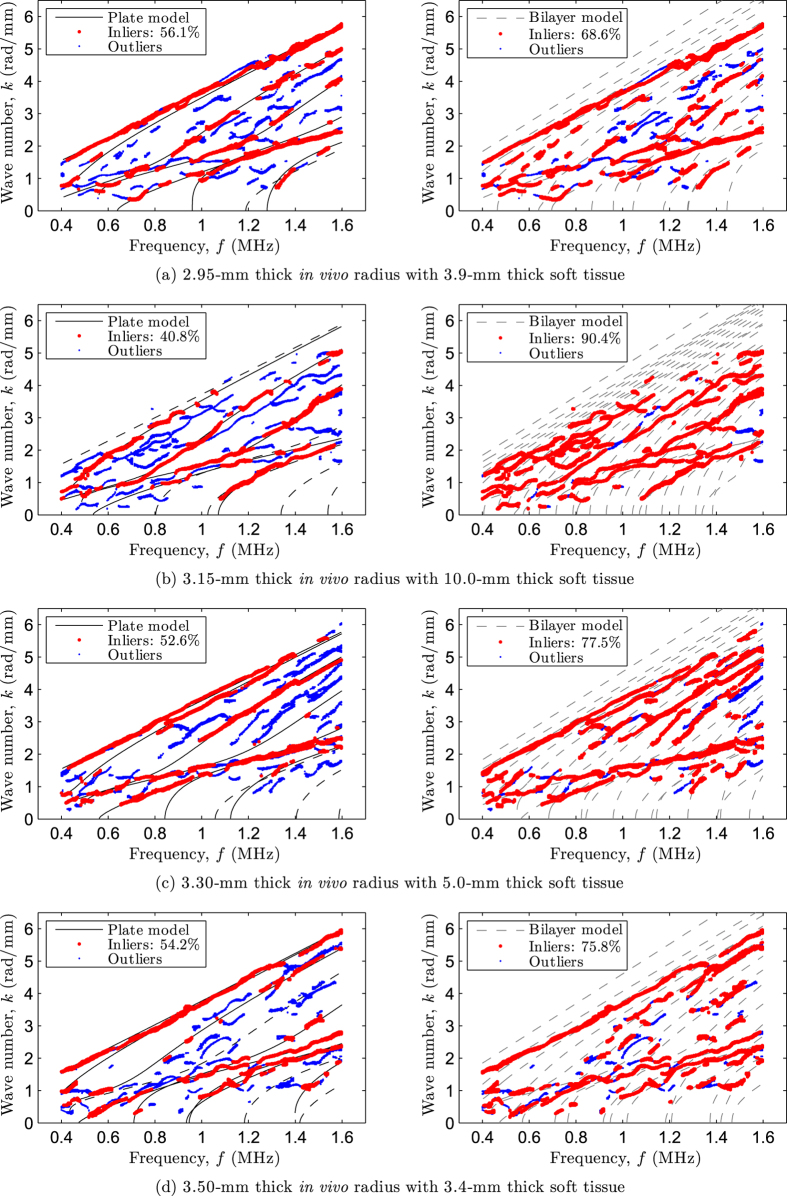
Matching between experimental data (dots) and theoretical modes (lines) for subjects with increasing cortical thickness: (**a**) 2.95 mm, (**b**) 3.15 mm, (**c**) 3.30 mm, and (**d**) 3.50 mm. Left plots represent the optimal matching resulting from the inverse problem solutions using the free plate model (modes that are missing in the optimal pairing vector ***M*** are displayed in discontinuous lines), whereas right plots represent the forward problem solutions calculated with the bilayer model.

Dispersion curves were measured *in vivo* on the four subjects. Figure 5 depicts the experimental data along with the theoretical modes for the four investigated cases. As for the bilayer phantoms, left plots show the inverse problem solutions using the free plate model, whereas right plots show the forward problem solutions using the bilayer model.

Again, the optimal inverse problem solutions also revealed that parts of the experimental data correspond to modes belonging to the cortical bone subsystem (matched by the free plate modes) despite the presence of soft tissue, irregular bone geometry, absorption and experimental noise. For the forward problem using the bilayer model, the acoustic properties of soft tissue were set to those of water, that are *c*_*f*_ = 1.54 mm·*μ*s^−1^ and *ρ*_*f*_ = 1 g·cm^−3^. The thickness of soft tissue for the four subjects was set to that measured with the 20 MHz pulse-echo technique, namely *h*_*f*_ = {3.9, 10.0, 5.0, 3.4} millimeters.

The ultrasound-based estimates of cortical thickness and bulk wave velocities, along with the reference thickness values delivered by the HR-pQCT measurements are summarized in Table 4.

Despite a lower signal-to-noise ratio and the presence of soft tissue, a good agreement was found between the experimental data and the free plate model. The relative differences on cortical thickness were lower than 5% and are in the order of magnitude of the precision of the reference method. For the sake of clarity, the estimates from the inversion procedure for the bulk wave velocities are also indicated in the table although they cannot be validated *in vivo*. We note, however, that these values are slightly higher that the range of values that can be derived from Table 1 through Eq. (1). In addition, the forward problem using the bilayer problem further supports the correctness of the estimates inferred with the free plate model, as most of the outliers from left plots are now adequately fitted (see the relative percentages of inliers). It is worth pointing out that, as for the bilayer phantoms with a tubular cross-section, the results suggest that *in vivo* measurements are less affected by the presence of soft tissue than coated plates.

## Discussion

In this study, we reported on the inversion of multimode GWs to investigate to which extent a 2-D transverse isotropic free plate model is accurate enough to recover reliable estimates of cortical bone properties, despite the presence of overlying soft tissue and bone curvature. Our two-steps approach, consisting of an inversion procedure using a 2-D transverse isotropic free plate model plus a forward problem with a 2-D free bilayer model, was first tested on a series of laboratory-controlled measurements performed on bone-mimicking plates and tubes coated with different soft tissue-mimicking layers, and then evaluated *in vivo* at the radius.

For the bilayer phantoms, correct thickness and stiffness estimates of the plates and tubes could be retrieved through the multiparametric inversion using the free plate model, even for relatively thick soft tissue-mimicking layers (~6–10 mm). Although the coatings induced the presence of additional guided modes and slightly modified the trajectory of modes propagating in the solid plate subsystem, the robust inversion procedure developed in ref. 24 demonstrated that parts of the experimental data still could be identified as free plate modes. In addition, the forward computation of a bilayer model fed with the inferred estimates (plus known soft tissue-mimicking properties) showed that such model was more precise to predict the experimental data, as the resulting model nearly perfectly matched the experimental data (i.e., around 75% of inliers) considered as outliers when using the free plate model. Nonetheless, although it predicted only parts of the data, a free plate model was precise enough to recover reliable estimates from the solid waveguide. As a by-product of the phantom study, it should be noted that our proposal encompassed several aspects that were not simultaneously considered so far (see Supplementary Table S1), namely: (i) the ratio between the thickness of the soft tissue-mimicking layer and that of the solid layer covered a wide range of values (i.e., *τ* = 0.5–8) that match realistic *in vivo* ratios at both the radius and tibia^15^,^39^, two skeletal sites that can actually be measured using axial transmission; (ii) the employed soft tissue-mimicking materials had acoustic properties close to those of real soft tissue (recall Table 2); and (iii) both phantoms with planar and tubular cross-section were investigated to account for the influence of curvature on the propagation characteristics of the bilayer guided modes.

For *in vivo* cortical bone, the thickness estimates were in good agreement with the reference values delivered by HR-pQCT measurements, whereas the stiffness estimates, which are indicated here only for the sake of completeness as outcomes of the inverse procedure, are slightly higher than the range of values for cortical bone reported in the literature (see Table 1). This slight difference can be explained by the fact that our measurements were carried out at the forearm of young healthy subjects, whereas literature values were measured in femur obtained from elderly donors. There are currently no available technologies to measure anisotropic elasticity *in vivo*. As for other ultrasound-based technologies, a surrogate for tissue elasticity cannot be directly validated *in vivo* but requires previous calibration on phantoms and *ex vivo* validation studies. Our approach was validated on phantoms, and, although its clinical value is beyond the scope of the present work, we believe that the *in vivo* prospective results presented here are of interest for the community and represent a first step towards clinical applications. To fully validate our stiffness estimates *ex vivo*, a face-to-face comparison between GWs-based characterization of bone specimens and resonant ultrasound spectroscopy measurements is currently ongoing. As a limitation, it should be noted that our approach was only tested *in vivo* on a small number of young healthy subjects, whose cortical thickness and stiffness cover a range that may likely differ from patients at risk of fractures, in whom thinner cortex, impaired mechanical properties^40^ and/or thicker soft tissue are expected^15^. Further studies should thus be extended to investigate a larger number of subjects, in whom bone properties cover a broader range that might be associated with aging and pathologies. The latter could lead to irregular inner cortical boundaries and larger pores, which may challenge the hypothesis that cortical bone behaves as a waveguide for ultrasound.

**Figure 6:**
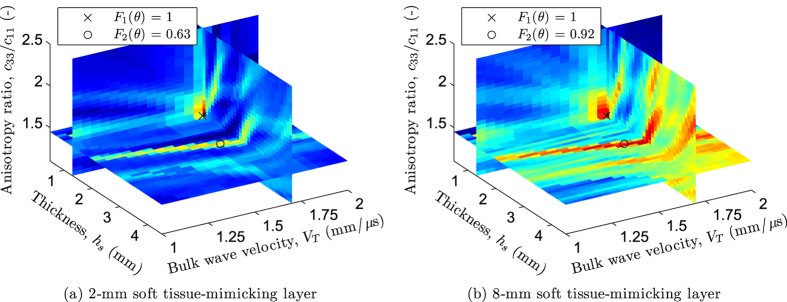
3-D slices of the normalized objective function *F(**θ***) along three model parameters, i.e., *h*_*s*_, *V*_*T*_ and *c*_33_/*c*_11_, with optimal values of the remaining three (*c*_13_/*c*_11_, 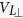 and ***M***): Example of a 1.25 mm bone-mimicking plate coated with (**a**) a 2 mm soft tissue-mimicking layer and (**b**) a 8 mm soft tissue-mimicking layer. The cross and the circle represent the global and a local optimum associated with the value of the objective function *F*_1_(***θ***) and *F*_2_(***θ***), respectively.

As a further limitation, it is worth pointing out that soft tissue thickness larger than 10 mm could cause the failure of our approach, as the inverse procedure could lead to an ill-posed problem with numerous local optima with similar values of the objective function. To support this statement, Fig. 6 depicts 3-D slices of the normalized objective function *F(**θ***) along three model parameters, i.e., *h*_*s*_, *V*_*T*_ and *c*_33_/c_11_, with optimal values of the remaining three (*c*_13_/*c*_11_, 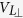 and ***M***), for the 1.25 mm bone-mimicking plate coated with a 2 mm and a 8 mm soft tissue-mimicking layer, respectively (see Fig. 3(a) and (d)). The intersection of these slices corresponds to the global optima (i.e., *F*_1_(***θ***) = 1). As can be observed, the relative amplitudes between global and local optima tend to decrease with increasing coatings thickness (i.e., *F*_2_(***θ***)). However, rather than a misidentification of the inverse procedure or a limitation of the plate model, such potential source of failure is mostly due to the poor quality of the measurements. Indeed, the difficulty of measuring GWs *in vivo* on subjects with high body mass index was previously reported^15^,^41^.

On the basis of earlier studies, this paper aimed at clarifying a potential source of confusion for the reader between the forward problem (i.e., prediction) and the inverse problem (i.e., inference), whose expected outcomes are different^42^. Indeed, the purpose of a waveguide model can be either (a) to represent an exact physical forward model or (b) to represent a simplified but sufficient inverse model to fit experimental data and provide a robust inference of one or more waveguide properties. An exact physical model should obviously take the soft tissue influence into account, as it should perhaps include the effects of curvature, anisotropy and some other additional factors. In such a case, the result is obvious that a free plate model predicts *in vivo* measurements in a rather incomplete manner, and one could intuitively expect that a more complex model^12^,^13^,^15^,^19^ might be necessary to perform an accurate inversion of such data. This does not mean, however, that using a free plate model is not adequate to inverse *in vivo* data, as its purpose is not exactly to accurately reproduce all the data, but only to provide a means to infer characteristics from a simplified *equivalent* system that might be linked to the real characteristics of the medium. In fact, the dispersion curves measured *in vivo* feature regions of the *f*−*k* plane that exhibit (i) the fingerprints of the cortical bone subsystem, (ii) the fingerprints of the soft tissue subsystem and (iii) the impact of coupling between the two subsystems. There are different axial transmission measurement setups that show off different sensitivities to these different regions, depending on the frequency bandwidth and some other factors that are not fully understood yet. In the context of multimode GWs measurements performed with a large frequency-thickness product range, our experimental data mainly exhibit sensitivity to the influence of the solid subsystem (i.e., bone), in which case a free plate model provides an appropriate inverse model. Consequently, our approach does not rule for or against the validity of the free plate model in its forward use. However, it demonstrates the adequacy of such a simplified model to solve the inverse problem. The high level of consistency between the reference values and the inferred properties *a posteriori* justifies our approach.

Furthermore, although a bilayer model has been shown to be a valuable predictive tool in different nondestructive evaluation applications, including, for instance, the material characterization of thin coatings^43^,^44^,^45^ or the application of piezoelectric materials to the problem of physical and chemical liquid sensing^46^,^47^, we believe that it raises several concerns for the *in vivo* assessment of cortical bone properties.

As a first limitation, it is challenging to solve a multiparametric inverse problem involving a bilayer model for at least three reasons. First, in the case of a transverse isotropic solid waveguide, such model depends upon nine model parameters. Beside the five model parameters of the solid waveguide (i.e., *c*_13_/*c*_11_, *c*_33_/*c*_11_, 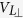, *V*_*T*_ and *h*_*s*_) and the three model parameters involving the soft tissue properties (i.e., *κ*_*f*_, *τ* and *γ*), the mass density *ρ*_*s*_ of the solid waveguide, which is typically unknown for bone, must be explicitly prescribed to solve the dispersion equation of a bilayered system, whereas it can be embedded in the velocity parameters for that of the free plate model^11^. Second, as experimental dispersion curves are usually incomplete and noisy, solving the inverse problem may lead to an ill-posed inversion (i.e., numerous local optima) and overfitting of the data. For example, as depicted in Fig. 4(d) (right plot), the number of modes (*n* = 28) is so large that any bilayer model would probably match the data. Third, solving the inverse problem using a global search algorithm (e.g., genetic algorithms) requires numerous evaluations of the forward problem, whose computational cost highly depends upon the model complexity. Indeed, to avoid numerical instability that may arise from the underlying optimization algorithm (e.g., Newton-Raphson) for computing the roots of the dispersion equation for extracting spatially-close modes, small wave numbers steps are needed for predicting the corresponding frequency^48^. Consequently, the forward calculation of the bilayer model has a larger computational cost than the plate model (i.e., a single forward problem requires around 0.4 and 8 seconds for the free plate and bilayer model, respectively).

As a further drawback, although the physical reasons are not established yet (e.g., geometry, nature of the coupling, etc.), the number of additional modes observed in our measurements, that is the impact of soft tissue, decreases for increasing waveguide complexity (i.e., plate - tube - cortical bone). Consequently, following the postulate of the *Ockham’s razor*^49^, the latter experimental evidence suggests that the simplest model (e.g., a free plate model) that is consistent with the data should be favored through a healthy balance between the information gained from the data (accuracy of the estimates) and the average goodness of fit (i.e., matching between the model and the data)^50^.

Future works should address novel ways for improving the extraction of experimental data belonging to the cortical bone subsystem and minimizing the impact of soft tissue, rather than the model complexity or the inversion scheme. In that vein, photo-acoustic imaging combined with a GWs-based approach has recently shown promising results on phantoms to focus the measurements at the interface between bone and soft tissue^18^. Alternatively, our group is currently taking advantage of the radio-frequency signals to obtain a real-time estimation of the soft tissue thickness, which could subsequently be used, prior to the inversion with a free plate model, to remove data belonging to soft tissue in parts of the dispersion curves corresponding to low phase velocities^12^, thus improving the accuracy of the ultrasound-based estimates of the solid waveguide. In that sense, a hybrid model combining plate modes plus a few low-order bilayer modes could also provide enhanced results, without altering too much the complexity of the inversion scheme.

## Additional Information

**How to cite this article:** Bochud, N. *et al*. Predicting bone strength with ultrasonic guided waves. *Sci. Rep.* **7**, 43628; doi: 10.1038/srep43628 (2017).

**Publisher's note:** Springer Nature remains neutral with regard to jurisdictional claims in published maps and institutional affiliations.

## Supplementary Material

Supplementary Information

## Figures and Tables

**Table 1:** Reference stiffness coefficients and mass density for the bone-mimicking samples and range for cortical bone properties.

	Stiffness coefficients (GPa)				Mass density (g · cm^−3^)
	*c*_11_	*c*_33_	*c*_13_	*c*_55_	*ρ*_*s*_
Bone-mimicking samples	14.7 ± 0.7	22.1 ± 0.9	7.6 ± 0.3	4.6 ± 0.2	1.65 ± 0.05
Cortical bone^27^,^28^,^29^	13.1–21.5	24.8–32.4	—	4.7–6.4	1.83–2.01

**Table 2:** Acoustic and structural properties of the soft tissue-mimicking materials (*Culjat *et al*. 51; **Values from 20 MHz pulse-echo measurements).

Material	Wave velocity	Mass density	Attenuation at 1 MHz	Thickness
	*c*_*f*_ (mm · *μ*s^−1^)	*ρ*_*f*_ (g · cm^−3^)	*α*_*f*_ (dB · cm^−1^)	*h*_*f*_ (mm)
Zerdine	1.54	1.03	0.52	2−10
Urethane	1.43	1.00	0.90	
Soft tissue	1.43–1.60*	0.95–1.05*	0.5–1*	3–10**

**Table 3:** Bilayer phantoms: ultrasound-based estimates of thickness and bulk wave velocities (plus relative errors with respect to the reference values), inferred using a free plate model (labels U and Z stand for Urethane and Zerdine, respectively).

Solid	Coating	Optimal model parameters 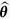				Reference thickness (mm)
		Bulk wave velocities (mm · *μ*s^−1^)			Thickness (mm)	
		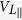	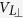	*V*_*T*_	*h*_*s*_	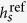
Plate 1	U: 2 mm	3.55 (3%)	2.96 (1%)	1.70 (2%)	1.28 (2%)	**1.25** ± **2%**
	Z: 4 mm	3.63 (1%)	2.90 (3%)	1.68 (1%)	1.28 (2%)	
	U: 6 mm	3.58 (2%)	2.98 (0%)	1.75 (5%)	1.31 (5%)	
	Z: 8 mm	3.52 (4%)	2.90 (3%)	1.78 (**7%**)	1.25 (0%)	
Plate 2	U: 2 mm	3.72 (2%)	2.92 (2%)	1.63 (2%)	2.51 (7%)	**2.34** ± **2%**
	Z: 4 mm	3.67 (0%)	3.15 (6%)	1.67 (0%)	2.43 (4%)	
	U: 6 mm	3.54 (3%)	2.98 (0%)	1.58 (5%)	2.40 (3%)	
	Z: 8 mm	3.83 (5%)	3.11 (4%)	1.52 (**9%**)	2.39 (2%)	
Plate 3	U: 2 mm	3.71 (1%)	2.99 (0%)	1.52 (**9%**)	3.56 (2%)	**3.48** **±** **6%**
	Z: 4 mm	3.54 (3%)	2.97 (0%)	1.69 (1%)	3.45 (1%)	
	U: 6 mm	3.60 (2%)	3.10 (4%)	1.75 (5%)	3.60 (3%)	
	Z: 8 mm	4.00 (**9%**)	3.20 (**7%**)	1.67 (0%)	3.61 (4%)	
Tube 1	U: 2 mm	3.63 (1%)	3.12 (5%)	1.73 (4%)	2.49 (7%)	**2.32** **±** **3%**
	Z: 4 mm	3.58 (2%)	3.05 (2%)	1.57 (6%)	2.43 (5%)	
	U: 6 mm	3.65 (0%)	3.12 (5%)	1.68 (1%)	2.48 (7%)	
	U: 10 mm	3.69 (1%)	3.03 (2%)	1.66 (1%)	2.45 (6%)	
Tube 2	U: 2 mm	3.45 (6%)	2.95 (1%)	1.68 (1%)	3.41 (7%)	**3.66** ± **2%**
	Z: 4 mm	3.56 (3%)	3.06 (3%)	1.70 (2%)	3.87 (6%)	
	U: 6 mm	3.50 (4%)	2.89 (3%)	1.57 (6%)	3.73 (2%)	
	U: 10 mm	3.67 (0%)	3.02 (1%)	1.50 (**10%**)	3.44 (6%)	
References derived from Table 1 (row 1)		**3.66** **±** **5%**	**2.98** **±** **6%**	**1.67** **±** **5%**	**—**	**—**

**Table 4:** Subjects: ultrasound-based estimates of thickness and bulk wave velocities (plus relative errors with respect to the reference thickness), inferred using a plate model.

	Optimal model parameters 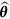				Reference HRpQCT-thickness (mm)
	Bulk wave velocities (mm · μs^−1^)			Thickness (mm)	
	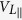	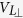	*V*_*T*_	*h*_*s*_	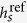
*In vivo* 1	4.12	3.43	1.85	2.89 (2%)	**2.95** ± **0.20**
*In vivo* 2	4.33	3.40	1.77	3.31 (5%)	**3.15** ± **0.20**
*In vivo* 3	4.07	3.43	1.82	3.24 (2%)	**3.30** ± **0.20**
*In vivo* 4	4.09	3.46	1.75	3.70 (5%)	**3.50** ± **0.20**
Ranges derived from Table 1 (row 2)	**3.68–4.02**	**2.68–3.27**	**1.60–1.78**	—	—

## References

[b1] Haentjens P. . Meta-analysis: excess mortality after hip fracture among older women and men. Ann. Intern. Med. 152, 380–390 (2010). 20231569 10.1059/0003-4819-152-6-201003160-00008 PMC3010729

[b2] Holzer G., von Skrbensky G., Holzer L. A. & Pichl W. Hip fractures and the contribution of cortical versus trabecular bone to femoral neck strength. J. Bone Miner. Res. 24, 468–474 (2009). 19016592 10.1359/jbmr.081108

[b3] Zebaze R. M. D. Intracortical remodelling and porosity in the distal radius and post-mortem femurs of women: a cross-sectional study. The Lancet 375, 1729–1736 (2010). 10.1016/S0140-6736(10)60320-0 20472174

[b4] Bala Y. . Cortical porosity identifies women with osteopenia at increased risk for forearm fractures. J. Bone Miner. Res. 29, 1356–1362 (2014). 24519558 10.1002/jbmr.2167 PMC4156822

[b5] Bala Y., Zebaze R. & Seeman E. Role of cortical bone in bone fragility. Curr. Opin. Rheumatol. 27, 406–413 (2015). 26002033 10.1097/BOR.0000000000000183

[b6] Nicholson P. H. F., Moilanen P., Kärkkäinen T., Timonen J. & Cheng S. Guided ultrasonic waves in long bones: modelling, experiment and *in vivo* application. Physiol. Meas. 23, 755–768 (2002). 12450274 10.1088/0967-3334/23/4/313

[b7] Lefebvre F., Deblock Y., Campistron P., Ahite D. & Fabre J. J. Development of a new ultrasonic technique for bone and biomaterials *in vitro* characterization. J. Biomed. Mater. Res. 63, 441–446 (2002). 12115753 10.1002/jbm.10261

[b8] Moilanen P., Nicholson P. H. F., Kilappa V., Cheng S. & Timonen J. Assessment of the cortical bone thickness using ultrasonic guided waves: Modelling and *in vitro* study. Ultrasound Med. Biol. 33, 254–262 (2007). 17306696 10.1016/j.ultrasmedbio.2006.07.038

[b9] Ta D., Wang W., Wang Y. Y., Le L. H. & Zhou Y. Measurement of the dispersion and attenuation of cylindrical ultrasonic guided waves in long bone. Ultrasound Med. Biol. 35, 641–652 (2009). 19153000 10.1016/j.ultrasmedbio.2008.10.007

[b10] Song X., Ta D. & Wang W. Analysis of superimposed ultrasonic guided waves in long bones by the joint approximate diagonalization of eigen-matrices algorithm. Ultrasound Med. Biol. 37, 1704–1713 (2011). 21924208 10.1016/j.ultrasmedbio.2011.06.028

[b11] Foiret J., Minonzio J.-G., Chappard C., Talmant M. & Laugier P. Combined estimation of thickness and velocities using ultrasound guided waves: A pioneering study on *in vitro* cortical bone samples. IEEE Trans. Ultrason. Ferroelect. Freq. Contr. 61, 1478–1488 (2014). 10.1109/TUFFC.2014.3062 25167148

[b12] Chen J. . Measurement of guided mode wavenumbers in soft tissue–bone mimicking phantoms using ultrasonic axial transmission. Phys. Med. Biol. 57, 3025–3037 (2012). 22538382 10.1088/0031-9155/57/10/3025

[b13] Tran T. N. H. T., Stieglitz L., Gu Y. J. & Le L. H. Analysis of ultrasonic waves propagating in a bone plate over a water half-space with and without overlying soft tissue. Ultrasound Med. Biol. 39, 2422–2430 (2013). 24035409 10.1016/j.ultrasmedbio.2013.06.007

[b14] Moilanen P., Nicholson P. H. F., Kilappa V., Cheng S. & Timonen J. Measuring guided waves in long bones: Modeling and experiments in free and immersed plates. Ultrasound Med. Biol. 32, 709–719 (2006). 16677930 10.1016/j.ultrasmedbio.2006.02.1402

[b15] Moilanen P. . Modeling the impact of soft tissue on axial transmission measurements of ultrasonic guided waves in human radius. J. Acoust. Soc. Am. 124, 2364–2373 (2008). 19062874 10.1121/1.2973228

[b16] Yapura C. L. & Kinra V. K. Guided waves in a fluid-solid bilayer. Wave Motion 21, 35–46 (1995).

[b17] Chen J. & Su Z. On ultrasound waves guided by bones with coupled soft tissues: A mechanism study and *in vitro* calibration. Ultrasonics 54, 1186–1196 (2014). 24008173 10.1016/j.ultras.2013.08.002

[b18] Moilanen P. . Photo-acoustic excitation and optical detection of fundamental flexural guided wave in coated bone phantoms. Ultrasound Med. Biol. 40, 521–531 (2014). 24361218 10.1016/j.ultrasmedbio.2013.10.018

[b19] Lee M.-Y., Yang C.-H. & Yang K. Modeling guided waves propagating in bones with a multilayered model. In IEEE Int. Ultrason. Symp. pages 761–764 (2014).

[b20] Minonzio J.-G. . A free plate model can predict guided modes propagating in tubular bone-mimicking phantoms. J. Acoust. Soc. Am. 137, EL98–EL104 (2015). 25618107 10.1121/1.4903920 PMC4277555

[b21] Vallet Q., Bochud N., Chappard C., Minonzio J.-G. & Laugier P. In vivo characterization of cortical bone using guided waves measured by axial transmission. IEEE Trans. Ultrason. Ferroelect. Freq. Contr. 63, 1361–1371 (2016). 10.1109/TUFFC.2016.2587079 27392349

[b22] Bossy E., Talmant M. & Laugier P. Three-dimensional simulations of ultrasonic axial transmission velocity measurement on cortical bone models. J. Acoust. Soc. Am. 115, 2314–2324 (2004). 15139643 10.1121/1.1689960

[b23] Protopappas V. C. . Three-dimensional finite element modeling of guided ultrasound wave propagation in intact and healing long bones. J. Acoust. Soc. Am. 121, 3907–3921 (2007). 17552737 10.1121/1.2354067

[b24] Bochud, N. et al. Genetic algorithms-based inversion of multimode guided waves for cortical bone characterization. *Phys. Med. Biol.* 61, 6953–6974 (2016). 10.1088/0031-9155/61/19/6953 27617648

[b25] Bernard S., Grimal Q. & Laugier P. Resonant ultrasound spectroscopy for viscoelastic characterization of anisotropic attenuative solid materials. J. Acoust. Soc. Am. 135, 2601–2613 (2014). 24815244 10.1121/1.4869084

[b26] Bernard S., Marrelec G., Laugier P. & Grimal Q. Bayesian normal modes identification and estimation of elastic coefficients in resonant ultrasound spectroscopy. Inverse Probl. 31, 065010 (2015).

[b27] Espinoza Orías A. A., Deuerling J. M., Landrigan M. D., Renaud J. E. & Roeder R. K. Anatomic variation in the elastic anisotropy of cortical bone tissue in the human femur. J. Mech. Behav. Biomed. Mater. 2, 255–263 (2009). 19627830 10.1016/j.jmbbm.2008.08.005 PMC2702870

[b28] Rudy D. J., Deuerling J. M., Espinoza Orías A. A. & Roeder R. K. Anatomic variation in the elastic inhomogeneity and anisotropy of human femoral cortical bone tissue is consistent across multiple donors. J. Biomech. 44, 1817–1820 (2011). 21543070 10.1016/j.jbiomech.2011.04.009 PMC3111152

[b29] Granke M. . Change in porosity is the major determinant of the variation of cortical bone elasticity at the millimeter scale in aged women. Bone 49, 1020–1026 (2011). 21855669 10.1016/j.bone.2011.08.002

[b30] Moreau L. . Accurate measurement of guided modes in a plate using a bidirectional approach. J. Acoust. Soc. Am. 135, EL15–EL21 (2014). 24437851 10.1121/1.4832335

[b31] Minonzio J.-G., Talmant M. & Laugier P. Guided wave phase velocity measurement using multi-emitter and multi-receiver arrays in the axial transmission configuration. J. Acoust. Soc. Am. 127, 2913–2919 (2010). 21117742 10.1121/1.3377085

[b32] Minonzio J.-G., Foiret J., Talmant M. & Laugier P. Impact of attenuation on guided mode wavenumber measurement in axial transmission on bone mimicking plates. J. Acoust. Soc. Am. 130, 3574–3582 (2011). 22225014 10.1121/1.3652884

[b33] Baron C., Talmant M. & Laugier P. Effect of porosity on effective diagonal stiffness coefficients (cii) and elastic anisotropy of cortical bone at 1 MHz: a finite-difference time domain study. J. Acoust. Soc. Am. 122, 1810–1817 (2007). 17927440 10.1121/1.2759165

[b34] Moreau L., Minonzio J.-G., Talmant M. & Laugier P. Measuring the wavenumber of guided modes in waveguides with linearly varying thickness. J. Acoust. Soc. Am. 135, 2614–2624 (2014). 24815245 10.1121/1.4869691

[b35] Rhee S.-H., Lee J.-K. & Lee J.-J. The group velocity variation of lamb wave in fiber reinforced composite plate. Ultrasonics 47, 55–63 (2007). 17881029 10.1016/j.ultras.2007.07.005

[b36] Yapura C. L. & Kinra V. K. Guided waves in a fluid-orthotropic solid bilayer. In Rev. Prog. Quant. Nondestr. Eval. pages 1633–1640. Springer (1997).

[b37] Chen J., Cheng L., Su Z. & Qin L. Modeling elastic waves in coupled media: Estimate of soft tissue influence and application to quantitative ultrasound. Ultrasonics 350–362 (2013). 22858152 10.1016/j.ultras.2012.06.018

[b38] Goldberg D. Genetic algorithms in search, optimization and machine learning. Addison-Wesley Publ., Reading, Massachussets (1989).

[b39] Kilappa V. . Low-frequency axial ultrasound velocity correlates with bone mineral density and cortical thickness in the radius and tibia in pre-and postmenopausal women. Osteoporosis Int. 22, 1103–1113 (2011). 10.1007/s00198-010-1273-7 20577874

[b40] Nishiyama K. K., Macdonald H. M., Buie H. R., Hanley D. A. & Boyd S. K. Postmenopausal women with osteopenia have higher cortical porosity and thinner cortices at the distal radius and tibia than women with normal abmd: an *in vivo* hr-pqct study. J. Bone Miner. Res. 25, 882–890 (2010). 19839766 10.1359/jbmr.091020

[b41] Talmant M. . *In vivo* performance evaluation of bi-directional ultrasonic axial transmission for cortical bone assessment. Ultrasound Med. Biol. 35, 912–919 (2009). 19243881 10.1016/j.ultrasmedbio.2008.12.008

[b42] Tarantola A. Inverse problem theory and methods for model parameter estimation. Society for Industrial and Applied Mathematics (2005).

[b43] Niklasson A. J., Datta S. K. & Dunn M. L. On approximating guided waves in plates with thin anisotropic coatings by means of effective boundary conditions. J. Acoust. Soc. Am. 108, 924–933 (2000). 11008796 10.1121/1.1286882

[b44] Simonetti F. Lamb wave propagation in elastic plates coated with viscoelastic materials. J. Acoust. Soc. Am. 115, 2041–2053 (2004).

[b45] Mezil S., Laurent J., Royer D. & Prada C. Non contact probing of interfacial stiffnesses between two plates by zero-group velocity lamb modes. Appl. Phys. Lett. 105, 021605 (2014).

[b46] Yang C.-H. & Shue C. J. Guided waves propagating in a piezoelectric plate immersed in a conductive fluid. NDT&E Int. 34, 199–206 (2001).

[b47] Wu C.-H. & Yang C.-H. Guided waves propagating in a bi-layer system consisting of a piezoelectric plate and a dielectric fluid layer. IEEE Trans. Ultrason. Ferroelect. Freq. Contr. 58, 1612–1618 (2011). 10.1109/TUFFC.2011.1988 21859580

[b48] Honarvar F., Enjilela E. & Sinclair A. N. An alternative method for plotting dispersion curves. Ultrasonics. 49, 15–18 (2009). 18727996 10.1016/j.ultras.2008.07.002

[b49] Gull S. F. Developments in maximum entropy data analysis. In Maximum entropy and Bayesian methods, pages 53–71, Springer (1989).

[b50] Bochud N. & Rus G. Probabilistic inverse problem to characterize tissue-equivalent material mechanical properties. IEEE Trans. Ultrason. Ferroelect. Freq. Contr. 59, 1443–1456 (2012). 10.1109/TUFFC.2012.2345 22828840

[b51] Culjat M. O., Goldenberg D., Tewari P. & Singh R. S. A review of tissue substitutes for ultrasound imaging. Ultrasound Med. Biol. 36, 861–873 (2010). 20510184 10.1016/j.ultrasmedbio.2010.02.012

