# Supplementary material for: Predicting bone strength with ultrasonic guided waves
Source: Sci Rep. 2017 Mar 3;7:43628. doi: 10.1038/srep43628 (PMC5335564; doi:10.1038/srep43628)
Supplement: Supplementary Information [file srep43628-s1.pdf]

Predicting bone strength with ultrasonic guided waves

Nicolas Bochud[1,*], **Quentin Vallet**[1], **Jean-Gabriel Minonzio**[1], **and Pascal Laugier**[1]

[1]Sorbonne Universités, UPMC Univ Paris 06, CNRS UMR 7371, INSERM UMR S1146, Laboratoire d'imagerie biomédicale, 15 rue de l'école de médecine, F-75006, Paris, France
[*]nicolas.bochud@espci.fr

ABSTRACT

Recent bone quantitative ultrasound approaches exploit the multimode waveguide response of long bones for assessing properties such as cortical thickness and stiffness. Clinical applications remain, however, challenging, as the impact of soft tissue on guided waves characteristics is not fully understood yet. In particular, it must be clarified whether soft tissue must be incorporated in waveguide models needed to infer reliable cortical bone properties. We hypothesize that an inverse procedure using a free plate model can be applied to retrieve the thickness and stiffness of cortical bone from experimental data. This approach is first validated on a series of laboratory-controlled measurements performed on assemblies of bone- and soft tissue mimicking phantoms and then on *in vivo* measurements. The accuracy of the estimates is evaluated by comparison with reference values. To further support our hypothesis, these estimates are subsequently inserted into a bilayer model to test its accuracy. Our results show that the free plate model allows retrieving reliable waveguide properties, despite the presence of soft tissue. They also suggest that the more sophisticated bilayer model, although it is more precise to predict experimental data in the forward problem, could turn out to be hardly manageable for solving the inverse problem.

Supplementary Introduction

Clinical applications using GWs remain, however, challenging for assessing multiple bone biomarkers (e.g., cortical thickness, stiffness and porosity), as the impact of the soft tissue layer on top of bone is not well established yet. Indeed, this fluid-like layer is expected to modify the trajectory of the dispersion curves and the relative intensities of guided modes belonging to the solid waveguide[16], which may bias the estimates of these biomarkers. A recent study by our group reported the first *in vivo* estimates of cortical thickness in healthy subjects using multimode GWs measurements [21]. Nonetheless, this study assumed a fixed elasticity and only considered higher-order modes (i.e., phase velocity higher than 3 mm$\cdot\mu$s^{-1}) to avoid the influence of soft tissue. To date, the influence of overlying soft tissue has only been analyzed in a few phantom studies, which can be classified in two categories according to the investigated frequency-thickness ($f - h_s$) product (where h_s denotes the thickness of the solid substrate). For equivalent thickness ranges, works of the first category achieved low frequency measurements of low-order modes, focusing on the fundamental flexural guided wave (FFGW) (equivalent to the A_0 Lamb mode for a plate)[14,15,17,18], whereas works of the second category carried out multimode GWs measurements using a higher driving frequency[12,13,19,20].

Among the first category, some studies, using fluid-solid bilayer plate or tube models, suggested that the identification of the FFGW may be challenging *in vivo*, because the modes propagating in soft tissue may be mistaken with those propagating in bone, i.e., the velocity of the FFGW (1.3 to 2.0 mm·μs^{-1}) overlaps that of soft tissue (1.4 to 1.6 mm·μs^{-1}). Nonetheless, results suggested that the measurement of the FFGW could be possible on some patients, as long as the thickness of the overlying soft tissue remains lower than 3 millimeters[14]. In a later related numerical study, an increasing mode density was reported for increasing soft tissue thickness[15]. It also showed an impact of soft tissue on the trajectory of the FFGW at low frequencies (less than 0.35 MHz), when its phase velocity is close to that of soft tissue. However, the extrapolation of this observation to a higher frequency range and higher-order modes is not straightforward.

Among the second category, Chen et al[12], assuming that a fluid-solid bilayer waveguide consisting of a soft tissue-mimicking layer (e.g., water, silicon and glycerol) on top of a bone-mimicking plate can be modeled as two independent waveguides (under the hypothesis that the mass densities of the fluid and solid layers are significantly different), showed that the higher-order modes propagating in the coated bone-mimicking plate were in good agreement with their counterparts in the free bone-mimicking plate. Consequently, they suggested that, by properly eliminating the modes belonging to the soft tissue layer, it might then be conceivable to recover bone properties by carrying out an inversion scheme comparing the remaining modes to a free plate model. In that vein, Minonzio et al[20], in an experimental study of the effect of a viscoelastic soft tissue-mimicking coating on top of tubular bone-mimicking phantoms, showed that the thickness and stiffness of the tubes could be properly estimated by inverting a free plate model in the considered bandwidth (i.e., $f \cdot h_s > 0.4$ MHz·mm), despite the presence of the soft tissue layer and the sample curvature. Nonetheless, it should be noted that the properties of the employed silicon rubber coating (i.e., velocity of 1.23 mm·μs^{-1} and thickness of 2.5 mm), which significantly differ from those of soft tissue, only induced a low interaction with the dispersion curves of the bone-mimicking phantoms. Alternatively, the effect of soft tissue on GWs was investigated on a bovine bone plate over a water half-space and overlaid by a gelatin layer using a solid-solid bilayer plate model[13]. An increase in modes number was observed, but the guided modes propagating in the bone plate, in particular the A_0 Lamb mode, were minimally affected by the addition of the soft tissue mimic.

To summarize, some common conclusions that can be drawn from these studies are that (i) the presence of soft tissue attenuates the time-domain signals, (ii) the number of modes significantly increases with increasing soft tissue thickness and (iii) the dispersion curves belonging to the solid waveguide are only modified in a certain range of the $f-k$ plane. It has also been shown that the use of sophisticated models (e.g., uncoupled bilayer[12], fluid-solid

bilayer[15], solid-solid bilayer[13], three-layered system[19]) can explain additional data arising from the presence of soft tissue. These studies, however, only solved the forward problem using *a priori* known bone-mimicking properties, without providing any hints on a possible inversion, i.e., on the ability to infer properties of cortical bone for clinical purposes. It is not clear yet that such sophisticated models could be used *in vivo*, where both cortical bone and soft tissue properties are unknown, and experimental data are noisy and incomplete. In addition, none of these studies except[12,19] considered bone as transverse isotropic, whereas it is expected that anisotropy has a significant influence for recovering reliable estimates of cortical bone properties[22,23]. Table S1 summarizes the materials and methods employed in the aforementioned studies to account for the impact of soft tissue.

	Authors	Materials					Waveguide model		Inverse problem	
		Bilayer types		Realistic properties						
		Phantoms	*In vivo*	Solid	Fluid	τ	Plate/tube model	Bilayer model	Thickness	Elasticity
FFGW	Moilanen et al, (2006)[14]	×						×		
	Moilanen et al, (2008)[15]	×	×		×	×		×		
	Chen et al, (2014)[17]	×						×		
	Moilanen et al, (2014)[18]	×				×		×		
Multimode GWs	Chen et al, (2012)[12]	×		×				×		
	Tran et al, (2013)[13]	×			×			×		
	Lee et al, (2014)[19]	×		×				×		
	Minonzio et al, (2015)[20]	×					×		×	×
	Vallet et al, (2016)[21]	×	×	×	×	×	×		×	
	Present study	×	×	×	×	×	×	×	×	×

Table S1. *Materials and methods employed in the literature to test the impact of soft tissue on the propagation characteristics of GWs (the variable $\tau = h_f/h_s$ denotes the ratio between the fluid and solid phase thickness).*

Supplementary Results

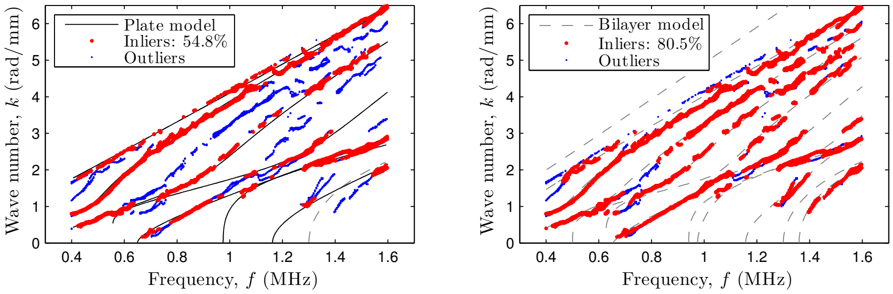

(a) 2.34-mm bone-mimicking plate coated with a 2-mm soft tissue-mimicking layer

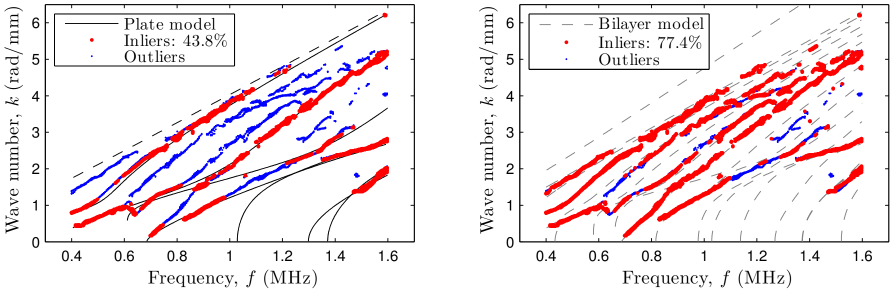

(b) 2.34-mm bone-mimicking plate coated with a 4-mm soft tissue-mimicking layer

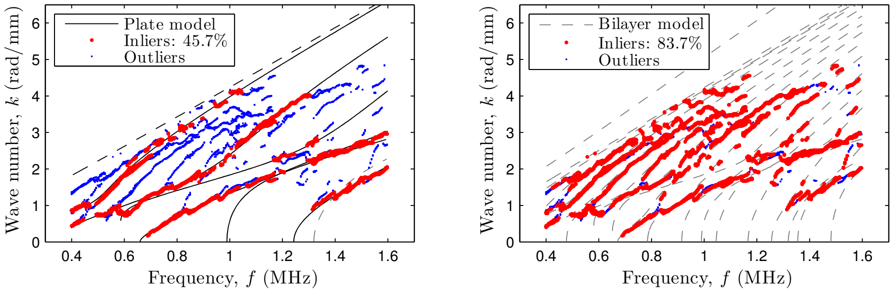

(c) 2.34-mm bone-mimicking plate coated with a 6-mm soft tissue-mimicking layer

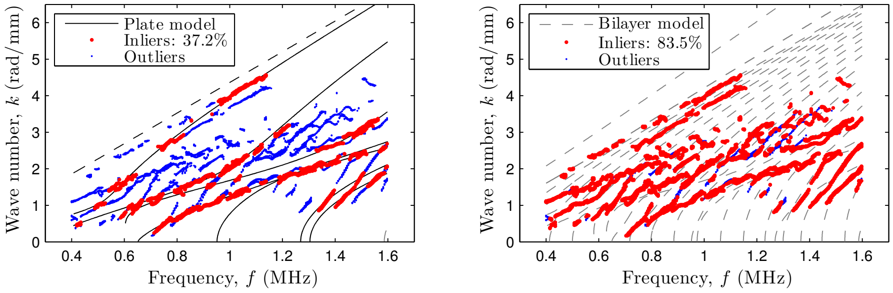

(d) 2.34-mm bone-mimicking plate coated with a 8-mm soft tissue-mimicking layer

Figure S1. *Matching between experimental data (dots) and theoretical modes (lines) for a 2.34-mm thick bone-mimicking plate coated with soft tissue-mimicking layers of increasing thickness: (a) 2-mm, (b) 4-mm, (c) 6-mm, and (d) 8-mm. Left plots represent the optimal matching resulting from the inverse problem solutions using the free plate model (modes that are missing in the optimal pairing vector* ***M*** *are displayed in discontinuous lines), whereas right plots represent the forward problem solutions calculated with the bilayer model.*

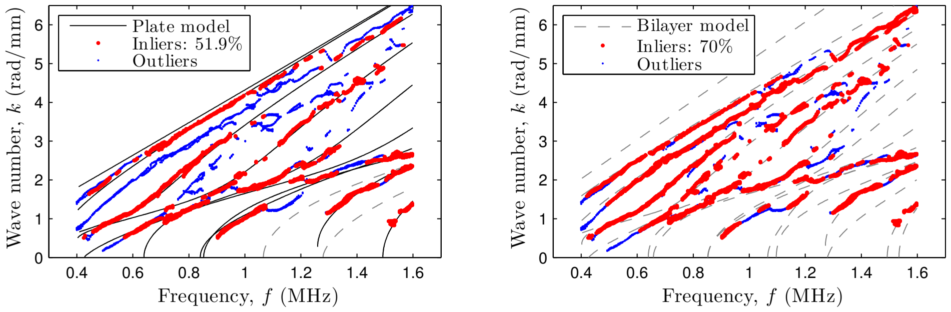

(a) 3.48-mm bone-mimicking plate coated with a 2-mm soft tissue-mimicking layer

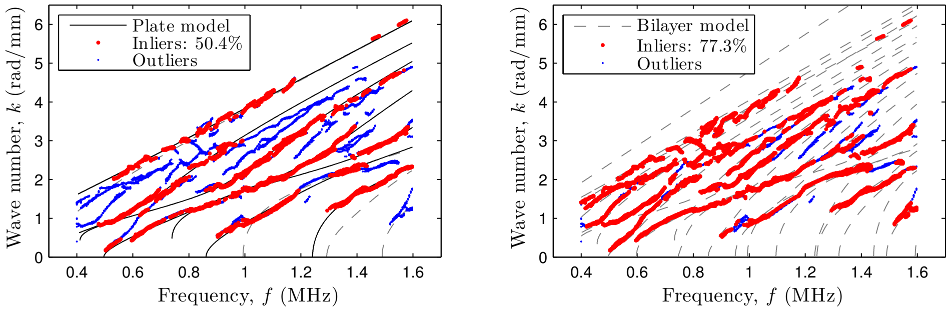

(b) 3.48-mm bone-mimicking plate coated with a 4-mm soft tissue-mimicking layer

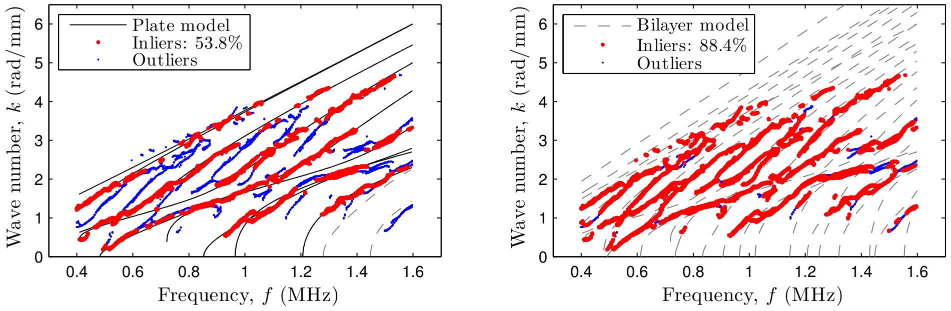

(c) 3.48-mm bone-mimicking plate coated with a 6-mm soft tissue-mimicking layer

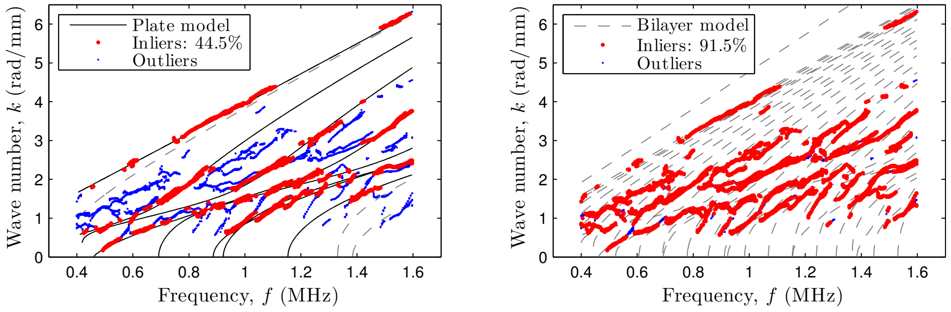

(d) 3.48-mm bone-mimicking plate coated with a 8-mm soft tissue-mimicking layer

Figure S2. *Matching between experimental data (dots) and theoretical modes (lines) for a 3.48-mm thick bone-mimicking plate coated with soft tissue-mimicking layers of increasing thickness: (a) 2-mm, (b) 4-mm, (c) 6-mm, and (d) 8-mm. Left plots represent the optimal matching resulting from the inverse problem solutions using the free plate model (modes that are missing in the optimal pairing vector **M** are displayed in discontinuous lines), whereas right plots represent the forward problem solutions calculated with the bilayer model.*

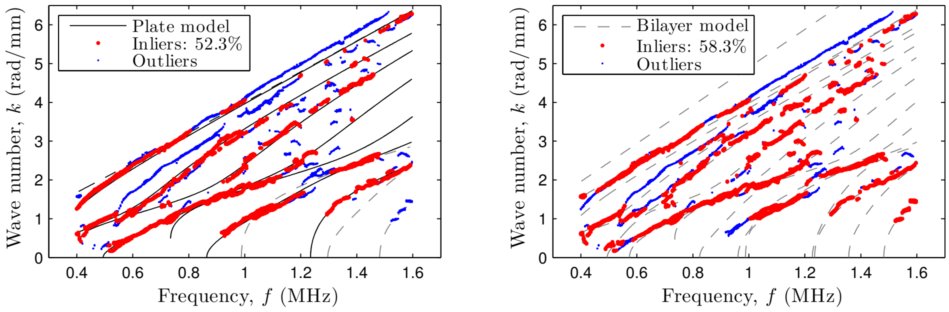

(a) 3.66-mm bone-mimicking tube coated with a 2-mm soft tissue-mimicking layer

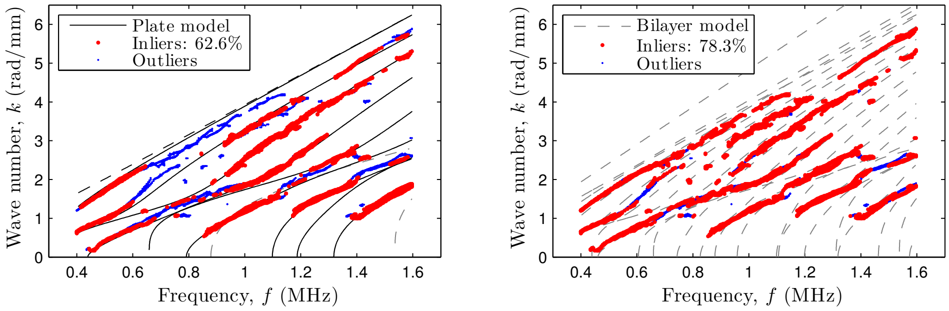

(b) 3.66-mm bone-mimicking tube coated with a 4-mm soft tissue-mimicking layer

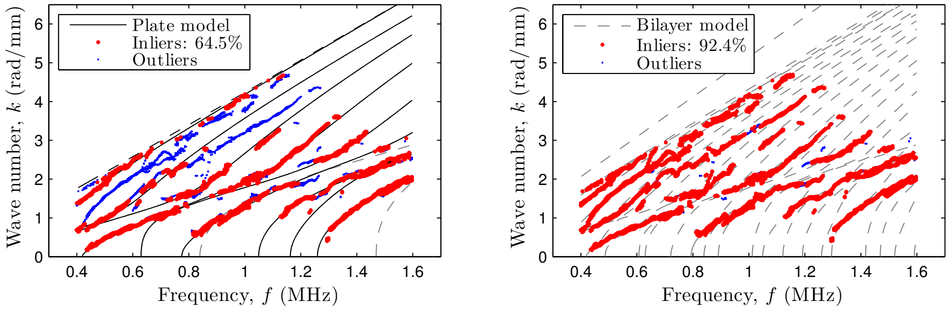

(c) 3.66-mm bone-mimicking tube coated with a 6-mm soft tissue-mimicking layer

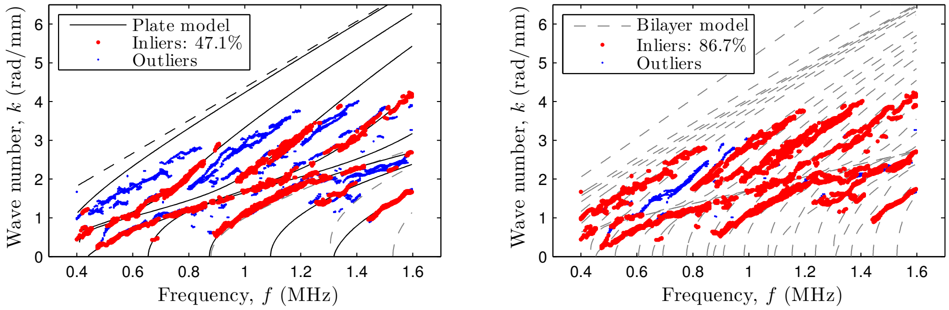

(d) 3.66-mm bone-mimicking tube coated with a 10-mm soft tissue-mimicking layer

Figure S3. *Matching between experimental data (dots) and theoretical modes (lines) for a 3.66-mm thick bone-mimicking tube coated with soft tissue-mimicking layers of increasing thickness: (a) 2-mm, (b) 4-mm, (c) 6-mm, and (d) 10-mm. Left plots represent the optimal matching resulting from the inverse problem solutions using the free plate model (modes that are missing in the optimal pairing vector* $\boldsymbol{M}$ *are displayed in discontinuous lines), whereas right plots represent the forward problem solutions calculated with the bilayer model.*
